# Hyperacute Superior Vena Cava Syndrome associated with Central Venous Catheter Insertion

**DOI:** 10.5005/jp-journals-10071-23140

**Published:** 2019-03

**Authors:** Stefan Edginton, Adam Fundytus

**Affiliations:** 1,2 Department of Internal Medicine, Queen's University, Kingston, Canada.

**Keywords:** Central venous catheters, Critical care, Dialysis catheter, Superior vena cava syndrome, Superior vena cava stenosis

## Abstract

Superior vena cava (SVC) syndrome is classically thought of as a complication of malignancy. However, SVC syndrome secondary to indwelling central venous catheters (CVCs) is another important entity. Amongst those with CVCs who develop SVC syndrome, the majority are attributed to thrombosis. Aside from thrombosis, CVCs can lead to SVC syndrome secondary to mechanical obstruction of blood flow in an already narrowed vessel.

We present the first case of hyperacute SVC syndrome that developed within 6 hours of insertion of a CVC into a patient's right internal jugular vein alongside a pre-existing right internal jugular tunnelled dialysis line. With removal of the line, the patient's symptoms resolved completely within hours. The patient also was found to have stenosis of superior vena cava, likely secondary to multiple instrumentations.

Physicians must be diligent to monitor for this complication in patients who have had previous instrumentations of major vessels when inserting CVCs.

**How to cite this article:**

Edginton S, Fundytus A. Hyperacute Superior Vena Cava Syndrome associated with Central Venous Catheter Insertion. Indian J Crit Care Med 2019;23(3):152-154.

## CASE REPORT

A 53-year old female was admitted to the Intensive Care Unit of Kingston General Hospital in Kingston, Ontario, Canada with diabetic ketoacidosis (DKA). She had a past medical history of type 1 diabetes with recurrent DKA and end stage renal disease secondary to diabetic nephropathy on intermittent hemodialysis. Prior to admission, she was being dialyzed through a left arm arteriovenous fistula.

Shortly after admission, her left arm fistula thrombosed, and a right internal jugular (IJ) tunneled double-lumen catheter was inserted for dialysis along with a peripherally inserted central catheter (PICC) via right basilic vein for intravenous access. The patient had a previous failed right arm AV fistula so the left arm was avoided in hopes of future recovery of the left AV fistula.

**Fig. 1:**
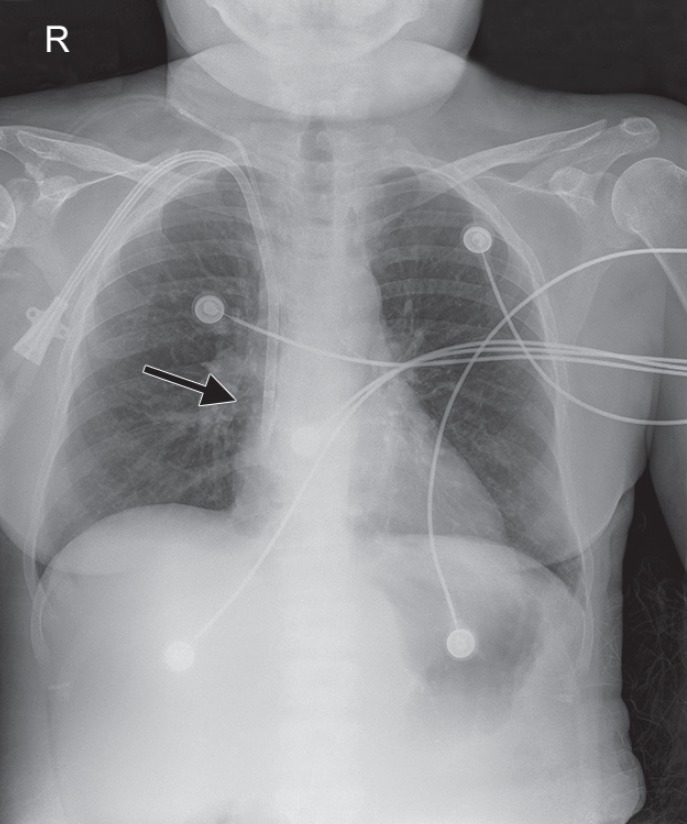
Chest X-ray post right internal jugular CVC insertion demonstrating proximity to right internal jugular dialysis catheter

Two weeks later during the course of her admission, she continued to have episodes of DKA, and inadvertently her PICC line was dislodged, requiring removal. The patient had poor peripheral intravenous (IV) access and multiple attempts to insert peripheral IVs under ultrasound guidance were unsuccessful. Ultrasound examination of the patient's left internal jugular showed significant narrowing. The patient declined an attempt femoral venous access because she had previous lower extremity venous grafting for her AV fistula and she was told to avoid cannulization of any of those vessels. A 7 French, 16cm triple-lumen CVC was placed into the right internal jugular vein with sonographic guidance without difficulty. The puncture site for the CVC insertion was significantly distal to the tunneled dialysis catheter. Post-procedure, all three lumens were able to draw blood and flush saline. A chest X-ray confirmed placement of the catheter adjacent to the tunneled dialysis catheter (Fig. 1) close to the cavoatrial junction. The patient did not have any immediate complaints or discomfort. Approximately six hours later, the on-call resident was called by nursing staff to assess the patient for new neck discomfort and jaw pain which had begun two hours prior.

**Figs 2A and B:**
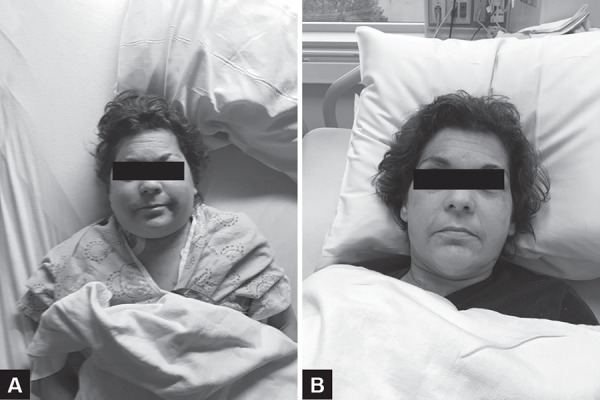
(A) Six hours after insertion of CVC. Patient has marked facial edema; (B) Six hours after CVC removed. Complete resolution of facial edema

At the time of assessment, the patient's heart rate was 95 beats per minute and regular. Her blood pressure was 188/84, respiratory rate was 18 and oxygen saturation was 99% on room air. She was afebrile. On examination, the patient had distended neck veins, pronounced facial edema and plethora (Fig. 2A). She also had bilateral upper-extremity edema primarily manifesting in her digits. The rest of her cardiorespiratory examination was unremarkable.

All three lumens of the CVC were withdrawing blood and flushing without difficulty.

Point of care ultrasound of the right internal jugular vein revealed no thrombus at the site of CVC entry or distal to it. The SVC was not visualized directly. The CVC was removed promptly. There was no visible clot on the catheter, and no clot was pulled along with the catheter. Within an hour, the patient's facial swelling and plethora began to resolve, and her neck pain began to improve. The next morning, pain and swelling had resolved completely (Fig. 2B).

**Fig. 3:**
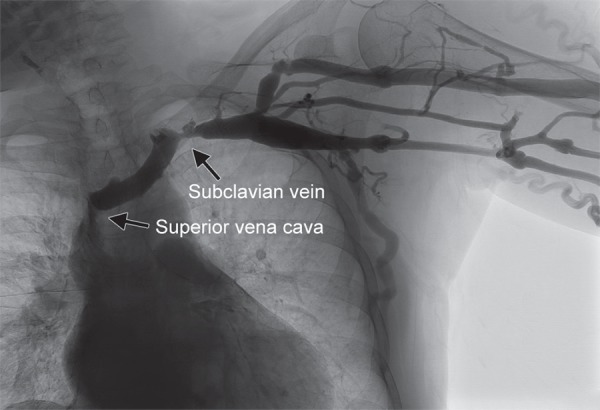
Fistulogram demonstrating narrowing of superior vena cava and left brachiocephalic vein

A retrospective chart review revealed a fistulogram performed months prior that showed significant stenosis at the left subclavian vein and mild narrowing at the junction where the two brachiocephalic veins merge to form the SVC (Figure 3). At that time, angiography was attempted to alleviate the stenosis. However, the patient did not tolerate this, and the procedure did not lead to a significant change in the degree of stenosis.

The events surrounding the SVC syndrome and the previously identified SVC stenosis were disclosed to the patient. In order to prevent a similar event from occurring in the future, a note was made in the chart that would be more visible should the need for further CVCs arise.

## Discussion

The SVC Syndrome is a clinical entity characterized by facial swelling in 82% of patients^1^. In the past, >90% of SVC syndromes were associated with malignancy^2^. However, contemporarily, roughly 40% of SVC syndromes are secondary to a benign etiology^1^. 71% of cases of non-malignant SVC syndrome have been attributed to intravascular devices, most commonly Port-a-Cath systems and dialysis catheters. The vast majority of benign SVC case reports in the literature report a thrombus associated with the indwelling line as the inciting factor^3–7^. The reported prevalence of central vein thrombosis with intravascular devices ranges from 5-42% while reports indicate that 1-14% of patients with these devices go on to develop SVC syndrome^1^. The majority of reported CVC-associated SVC syndromes in the literature manifested clinically between post- insertion day 1 to 8 and generally exhibited prolonged resolution phase^3–8^.

Our case is unique in that our patient presented with SVC syndrome within hours of insertion of a non-indwelling CVC and the symptoms resolved completely within hours of removal of the CVC. The SVC syndrome was likely caused by mechanical obstruction from the new CVC and the tunneled dialysis catheter in the setting of a smaller vessel diameter. This narrowing of vessels was likely secondary to repeated instrumentation and multiple intravascular devices.

The rapid onset of symptoms suggests the etiology was not a thrombus-mediated event. The patient had no predisposing factors for thrombosis such as active cancer or coagulopathy and she was receiving subcutaneous heparin prophylactically. Additionally, all lumens of the CVC were flushing and withdrawing well and there was no visible thrombus on the catheter after it had been withdrawn. The rapid and complete resolution of symptoms following removal of the line further supports this diagnosis.

There is one report in the literature describing a similar right IJ-mediated SVC syndrome, which developed in the setting of a stenotic SVC. However, the case developed over an unspecified time between hospital discharge and routine follow up^9^. Additionally, full resolution of symptoms took approximately 3 weeks^9^. There has also been one case report of a stenosis induced by the presence of both a Pacemaker and CVC within the superior vena cava that required angioplasty to relieve the SVC stenosis^5^.

While most cases of SVC syndrome related to CVCs are thrombus-mediated, our case brings to light a separate patient group that warrants clinical attention. In an era where both invasive cardiac devices and tunneled lines are common, clinically silent SVC stenosis at risk of flow compromise is likely to become more prevalent. It is important for clinicians to be aware of these risk factors as these patients are at a higher risk of developing iatrogenic CVC-associated SVC syndrome. Teams within a hospital dedicated to achieving difficult IV access may be helpful, as has been shown in a small study^10^. In patients with a history of multiple instrumentations of central veins, it is important to investigate for previously documented stenoses prior to insertion of new catheters and to monitor for these complications.
